# The *Mycobacterium tuberculosis* Proteasome Active Site Threonine Is Essential for Persistence Yet Dispensable for Replication and Resistance to Nitric Oxide

**DOI:** 10.1371/journal.ppat.1001040

**Published:** 2010-08-12

**Authors:** Sheetal Gandotra, Maria B. Lebron, Sabine Ehrt

**Affiliations:** Department of Microbiology and Immunology, Weill Cornell Medical College, New York, New York, United States of America; Harvard School of Public Health, United States of America

## Abstract

Previous work revealed that conditional depletion of the core proteasome subunits PrcB and PrcA impaired growth of *Mycobacterium tuberculosis in vitro* and in mouse lungs, caused hypersusceptibility to nitric oxide (NO) and impaired persistence of the bacilli during chronic mouse infections. Here, we show that genetic deletion of *prcBA* led to similar phenotypes. Surprisingly, however, an active site mutant proteasome complemented the *in vitro* and *in vivo* growth defects of the *prcBA* knockout (*ΔprcBA*) as well as its NO hypersensitivity. In contrast, long-term survival of *M. tuberculosis* in stationary phase and during starvation *in vitro* and in the chronic phase of mouse infection required a proteolytically active proteasome. Inhibition of inducible nitric oxide synthase did not rescue survival of *ΔprcBA*, revealing a function beyond NO defense, by which the proteasome contributes to *M. tuberculosis* fitness during chronic mouse infections. These findings suggest that proteasomal proteolysis facilitates mycobacterial persistence, that *M. tuberculosis* faces starvation during chronic mouse infections and that the proteasome serves a proteolysis-independent function.

## Author Summary

The eukaryotic proteasome is ubiquitous and essential for many basic cellular processes. In contrast to most bacteria, which do not express a proteasome, *Mycobacterium tuberculosis* encodes a proteasome predicted to be essential or required for optimal growth of the pathogen. Genetic silencing of the proteasome core genes further suggested that the *M. tuberculosis* proteasome plays an important role in defense against nitric oxide and in persistence of the pathogen during chronic mouse infections. In this manuscript we generated a genetic deletion mutant of the proteasome core genes proving that the 20S proteasome is not essential for growth of *M. tuberculosis*. We complemented the proteasome knockout with a proteolytically active and a mutated, proteolysis defective proteasome. This revealed that proteasomal proteolysis is dispensable for *in vitro* and *in vivo* growth and nitric oxide resistance of *M. tuberculosis* and suggests that the proteasome core serves a proteolysis-independent function. In contrast, long-term survival of the pathogen *in vitro* and in the chronic phase of mouse infection required a proteolytically active proteasome. We further provide evidence that nitric oxide is not responsible for killing of the proteasome knockout during chronic mouse infections. Thus, proteasomal proteolysis facilitates mycobacterial persistence independently of defense against nitric oxide. We propose that the failure to survive starvation contributes to the impaired persistence of *M. tuberculosis* lacking a proteolytically active proteasome during chronic infections.

## Introduction

Most cells continuously synthesize and degrade proteins in a regulated manner. Protein degradation is highly selective and this is achieved in part by localization of protease active sites within a barrel-shaped complex. This self-compartmentalization was first discovered for the proteasome [1], [2]. In all genera, the proteasome consists of a 20S cylindrical core particle, which contains two heptameric outer rings composed of α subunits, and two heptameric inner rings composed of the proteolytically active β subunits. The 20S proteasome belongs to the class of N-terminal nucleophile (Ntn) hydrolases, with a hydroxyl group of the amino-(N) terminal threonine functioning as catalytic nucleophile that reacts with peptide bonds of substrates or the electrophilic functional groups of proteasome inhibitors [3].

Bacterial proteasomes are only found in Actinomycetes [4], while other chambered proteases such as ClpAP, ClpXP, Lon, HslUV and FtsH are common in most bacteria [5], [6]. *Mycobacterium tuberculosis* encodes a proteasome and two CLP proteases, but lacks homologs of Lon and HslUV [7]. The proteasome accessory factors, *Mycobacterium* proteasomal ATPase (Mpa) and proteasome accessory factor A (PafA), are important for defense against reactive nitrogen intermediates (RNI) and for virulence of *M. tuberculosis* in the mouse [8]. Mpa assembles into a hexameric ATPase similar to the archeal proteasome associating nucleotidase (PAN) and the eukaryotic regulatory 19S cap [9], [10]. The *M. tuberculosis* 20S proteasome harbors electron dense plugs at the barrel ends created by the N-termini of its α subunits [11]. Removal of the N-terminal eight amino acids resulted in enhanced peptidolytic activity, suggesting that the *M. tuberculosis* proteasome has a gated structure and implying a role for accessory factors including Mpa in “gate opening” [9], [12], [13]. A direct interaction of purified Mpa with the 20S open gate mutant proteasome was demonstrated by electron microscopy [14].

In eukaryotic cells a covalently attached polymeric chain of ubiquitin targets proteins for degradation by the proteasome [15]. In *M. tuberculosis*, Pup, a prokaryotic ubiquitin-like protein, is ligated by PafA to proteasomal substrate proteins and serves as degradation signal [16], [17], [18]. Pup must be deamidated by Dop (deamidase of Pup) to activate it for conjugation to a substrate [16], [17], [18]. *In vitro* reconstitution assays with purified Dop, PafA, Pup, ATP and substrate proteins FabD (malonyl acyltransferase) or PanB (ketopantoate hydroxymethyltranferase) revealed that Dop and PafA are necessary and sufficient for *in vitro* pupylation of proteasome target proteins. Accordingly pupylation was severely impaired and PanB and FabD accumulated in an *M. smegmatis dop* deletion mutant [19]. Recently, the Mpa-proteasome complex has been reconstituted *in vitro* and shown to unfold and degrade Pup-tagged substrates via interaction of Mpa with Pup [20]. Interestingly Pup is degraded together with the substrate, in contrast to ubiquitin, which is recycled.

Numerous pupylated proteins of diverse cellular functions have been identified in *M. smegmatis* and *M. tuberculosis* [21], [22]. The overlap between nitrosylated and pupylated proteins suggests that the proteasome is important for turnover of nitrosylated proteins [22], [23]. This hypothesis is substantiated by hypersusceptibility to reactive nitrogen intermediates (RNI) of *M. tuberculosis* lacking proteasome associated factors or depleted for the proteasome core subunits PrcBA [8], [24]. However, it is unclear if accumulation of nitrosylated proteins or any other proteasome substrate(s) caused the growth and persistence defects of proteasome deficient *M. tuberculosis* in mouse lungs. To gain more insight into proteasome core function, we constructed a *prcBA* deletion mutant (*ΔprcBA*) and complemented it with either an active wild type core proteasome or a proteolytically defective, active site mutant proteasome. Our data suggest that proteasomal proteolysis is dispensable for *in vitro* and *in vivo* replication of *M. tuberculosis* and for resistance to RNI. Inhibition of inducible nitric oxide synthase (iNOS) did not affect killing of *ΔprcBA*, indicating that defense against NO is likely not a major activity by which the proteasome facilitates mycobacterial persistence. However, *M. tuberculosis* expressing the proteolysis defective proteasome was severely impaired in stationary phase survival, died in response to carbon starvation and failed to persist during chronic mouse infections. Thus, the *M. tuberculosis* proteasome may promote survival *in vivo* by opposing starvation.

## Results

### The 20S core proteasome is not essential but is required for optimal growth and resistance to nitric oxide

**Figure 1:**
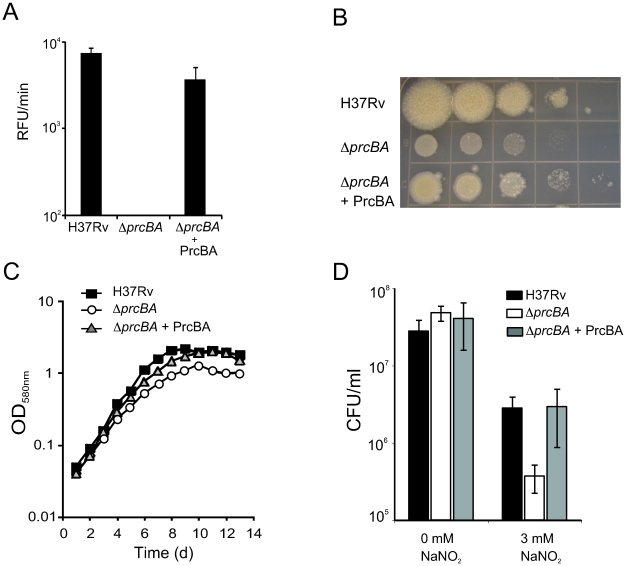
The proteasome is required for optimal growth *in vitro* and for resistance to RNI. (**A**) Proteasome activities in H37Rv, *ΔprcBA* and the complemented mutant (*ΔprcBA* + PrcBA). The cleavage velocity (RFU/min) of the fluorogenic peptide substrate Suc-LLVY-AMC reports proteasome activity. Proteasome activity was not detectable in *ΔprcBA*. Data are means ± s.d. of three independent experiments. (**B**) Growth of H37Rv, *ΔprcBA* and *ΔprcBA* + PrcBA on agar plates. Serial dilution of the three stains were spotted onto 7H11 agar plates and incubated for 3 weeks at 37°C. (**C**) Growth of H37Rv, *ΔprcBA* and *ΔprcBA* + PrcBA in liquid culture. Strains were inoculated in 7H9 media and growth was followed by measuring OD_580_. (**D**) Susceptibility to reactive nitrogen intermediates. Colony forming units (CFU) of H37Rv, *ΔprcBA* and the complemented strain after three days exposure to 0 mM and 3 mM sodium nitrite at pH 5.5. Data are means ± s.d. of triplicate cultures and representative of three independent experiments.

The genes encoding the *M. tuberculosis* proteasome core subunits PrcB and PrcA were predicted to be essential or required for optimal growth *in vitro* [25]. Conditional depletion of the proteasome core subunits via transcriptional silencing of *prcBA* resulted in impaired growth on agar plates and in liquid culture [24]. Here, we genetically deleted *prcBA* (Figure S1) resulting in loss of proteasome activity (Figure 1A). Expression of *prcBA* from a constitutive promoter on an episomal plasmid restored PrcB expression and proteasome activity in the complemented mutant (*ΔprcBA*+PrcBA) (Figure 1A, S1C). The *prcBA* knockout (*ΔprcBA*) was viable, yet impaired for growth on agar plates and had a small but reproducible growth defect in liquid culture, confirming previous observations (Figure 1B, C). The growth defects were restored in the complemented mutant. These data demonstrate that while the core proteasome is required for optimal growth of *M. tuberculosis in vitro*, it is not essential. The growth defect of *ΔprcBA* was more evident on agar plates than in liquid medium, similar to what we previously observed after *prcBA* silencing [24].

*M. tuberculosis* mutants that lack the mycobacterial proteasome ATPase Mpa or the Pup ligase PafA or are depleted for PrcBA are hypersusceptible to RNI [8], [24]. Similarly, viability of *ΔprcBA* was almost ten-fold reduced compared to wt *M. tuberculosis* after exposure to acidified sodium nitrite (Figure 1D). This increased killing was complemented when PrcBA were expressed from a plasmid. Thus, the 20 S proteasome core is required for resistance against RNI *in vitro*.

### The core proteasome is required for virulence in immune competent and immune compromised mice

**Figure 2:**
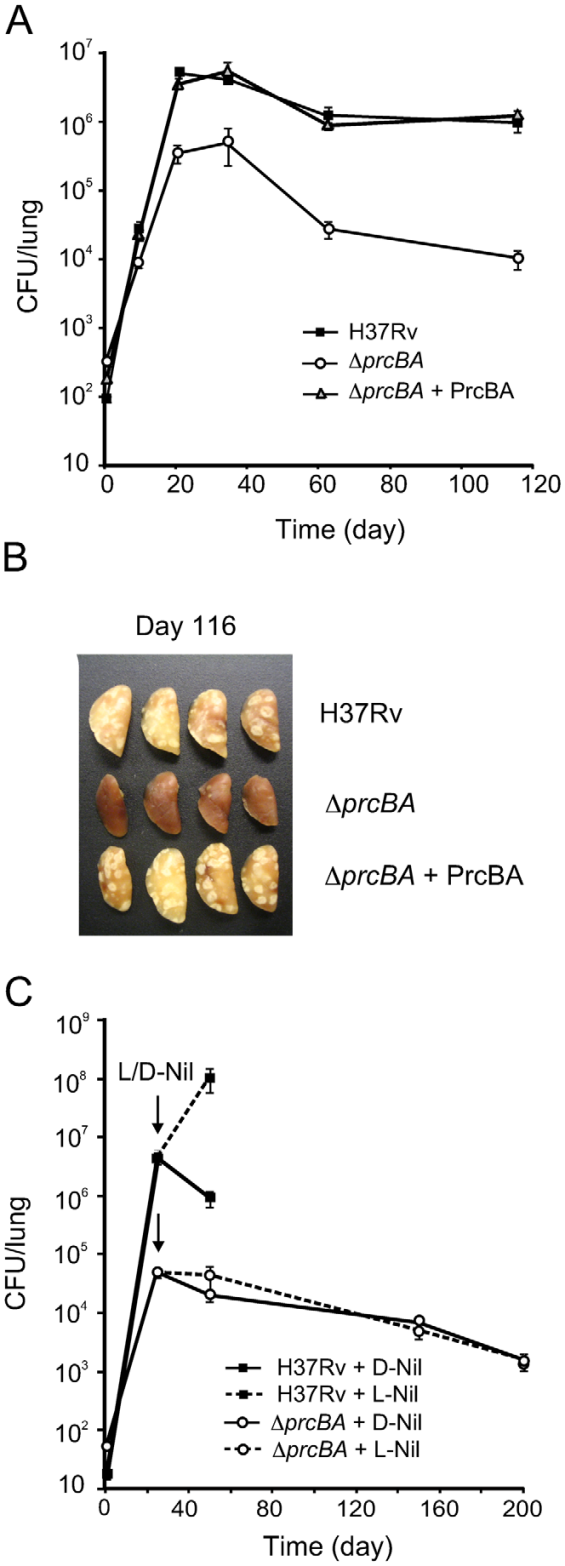
The proteasome is required for optimal growth and persistence in mice. (**A**) Bacterial titers in lungs of C57BL/6 mice infected by aerosol with H37Rv, *ΔprcBA* and the complemented mutant. Data are means ± s.d. from four mice per time point per group and are representative of three independent experiments. (**B**) Gross pathology of lungs infected with H37Rv, *ΔprcBA* and complemented mutant at day 116 post-infection. (**C**) Bacterial titers in lungs of C57BL/6 mice infected by aerosol with H37Rv and *ΔprcBA* and treated with the iNOS inhibitor L-Nil and its inactive enantiomer D-Nil starting at day 25 post-infection (indicated by arrow). Data are means ± s.d. from four mice per time point per group and represent two independent experiments.

The mouse model of tuberculosis is characterized by an acute phase, in which the bacteria replicate actively for approximately three weeks and a chronic phase, during which the bacteria persist at stable numbers. Silencing of *prcBA* reduced replication of *M. tuberculosis* during the acute phase and persistence during the chronic phase of infection in mouse lungs [24]. Genetic deletion of *prcBA* similarly affected *in vivo* growth and persistence of *M. tuberculosis* (Figure 2A, B). At three weeks post infection CFU in lungs were 1.5 log_10_ lower in *ΔprcBA* infected mice than in mice infected with wt *M. tuberculosis* and at 16 weeks post infection this difference increased to 2 log_10_ (*P* = 0.001 and *P* = 0.009). The virulence defects were fully restored in the complemented mutant. Nitric oxide generated by inducible nitric oxide synthase (iNOS) is required to control mycobacterial replication in mice [26] and lack of proteasome activity resulted in increased susceptibility of *M. tuberculosis* to RNI (Figure 1D). To determine if NO produced by iNOS was responsible for the decline in viability of *ΔprcBA* during the chronic phase of the infection, infected mice were treated with an iNOS-specific inhibitor L-*N^6^*-iminoethyl-lysine (L-NIL) [27], [28] starting at day 25 post infection (Figure 2C). In mice infected with wt *M. tuberculosis*, L-NIL treatment resulted in a failure to control bacterial replication, so that bacillary loads were increased by two orders of magnitude in lungs at 25 days post treatment (day 50 post infection) compared to the control group treated with the inactive enantiomer (D-NIL) (Figure 2C). The remaining L-NIL-treated mice infected with wt *M. tuberculosis* succumbed between day 50 and day 75 post-infection. In contrast, only a 2-fold increase in bacillary burden of *ΔprcBA* was observed in lungs upon L-NIL treatment compared to D-NIL treatment at 25 days post treatment. L-NIL treated mice infected with *ΔprcBA* survived until the end of the experiment (day 200), and bacterial numbers in the lungs of both L-NIL and D-NIL treated mice declined by 20-fold (Figure 2C). There was only a slight increase in the number of nodular lesions at day 200 in mice infected with *ΔprcBA* and treated with L- Nil compared to D-Nil treated mice (not shown). Altogether, these data suggest that iNOS is not required to control *ΔprcBA* during chronic infection in mice.

### Mutation of the PrcB active site residue threonine dramatically impairs proteolytic activity

**Figure 3:**
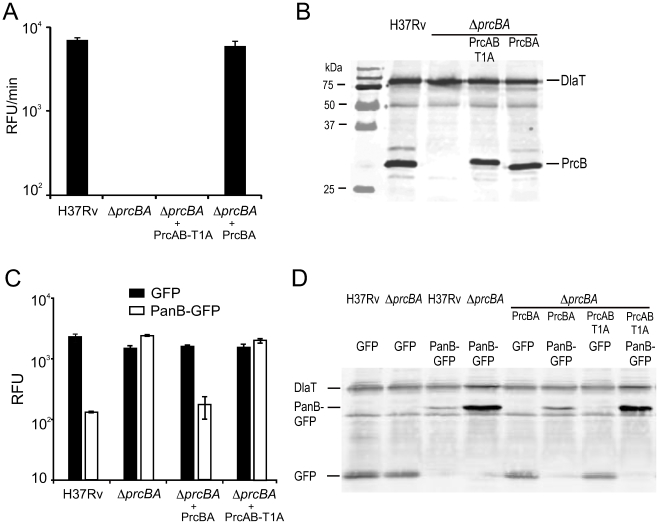
Characterization of the active site mutant proteasome. (**A**) Proteasome activities of H37Rv, *ΔprcBA* and *ΔprcBA* complemented with the active site mutant proteasome (PrcAB-T1A) and the intact proteasome (PrcBA) measured using the fluorogenic substrate Suc-LLVY-AMC. Proteasome activity was not detectable in *ΔprcBA* and *ΔprcBA* complemented with the active site mutant proteasome (PrcAB-T1A). (**B**) PrcB levels analyzed by immunoblotting in lysates from H37Rv, *ΔprcBA* and the complemented strains. DlaT was used as loading control. (**C**) GFP activities in strains expressing GFP or the PanB-GFP fusion protein. (**D**) GFP and PanB-GFP levels analyzed by immunoblotting in lysates from H37Rv, *ΔprcBA* and the complemented strains. DlaT was used as loading control.

The 20S proteasome is a multimeric protein complex and we hypothesized that lack of expression of the proteasome core subunits PrcB and PrcA could have different physiological consequences than lack of PrcB-mediated proteolytic activity. To test this, we expressed a proteasome active site mutant in *ΔprcBA*. In this mutant, the N-terminal threonine residue of the mature PrcB subunit was mutated to alanine (T1A). This mutation abolished proteolytic activity of the proteasome from *Thermoplasma acidophilum* [3], [29], [30]. To allow assembly of this mutant proteasome subunit into a 20S complex, we also deleted the pro-peptide of the PrcB subunit, which in an active proteasome is autocatalytically removed by the active site Thr, thereby exposing the amino group of Thr for nucleophilic attack on the target peptide bond [12], [29], [31]. Similar mutagenesis of the *T. acidophilum* proteasome allowed assembly of a mature 20S proteasome core [29], [31], [32]. The mutated *prcB* gene (*pcrB*T1A) was cloned including a C-terminal histidine tag in an operon with *prcA* (*prcAB*T1A) and expressed in *ΔprcBA*. Immunoprecipitation of the PrcB subunit from lysates of this *M. tuberculosis* strain co-purified PrcA as determined by liquid chromatography-tandem mass spectrometry (Figure S2) indicating that a complex containing both subunits formed *in vivo* despite the PrcBT1A mutation. As expected, the mutant proteasome failed to complement proteolytic activity of *ΔprcBA*, measured by cleavage of the peptide substrate Suc-LLVY-AMC (Figure 3A), although the expression level of the PrcB subunit containing the T1A mutation was similar to that of wt PrcB (Figure 3B). To determine whether the T1A mutation affected proteolytic activity within the bacteria, GFP was fused to the proteasome substrate PanB (ketopantoate hydroxymethyltransferase). PanB has been shown to accumulate in *M. tuberculosis* lacking Mpa and in wt *M. tuberculosis* treated with the proteasome inhibitor epoxomicin [33]. We confirmed by 2-D SDS page analysis that PanB also accumulated in *ΔprcBA* (not shown). Similarly, the PanB-GFP fusion protein accumulated in *ΔprcBA* compared to wt *M. tuberculosis* as shown by GFP activity (Figure 3C) and GFP protein levels (Figure 3D). This was complemented when the intact core proteasome (PrcBA) was expressed in *ΔprcBA*. In contrast, the active site mutant proteasome (PrcAB-T1A) did not revert the accumulation of PanB-GFP (Figure 3C, D). Thus, the active site mutation T1A not only abolished proteasome activity against a peptide substrate *in vitro*, but also disrupted proteolytic activity of the proteasome within the bacteria.

### The active site mutant proteasome enables optimal growth *in vitro* and *in vivo*, confers RNI resistance but is not sufficient for persistence in mice

**Figure 4:**
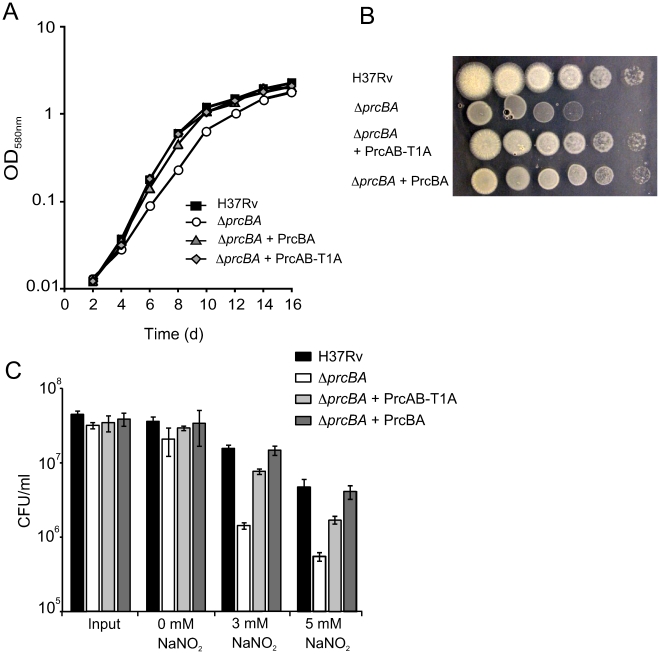
The active site mutant proteasome complements the growth defects and RNI hypersusceptibility of the proteasome KO. (**A**) Growth of H37Rv, *ΔprcBA* and the complemented strains in liquid culture. Strains were inoculated in 7H9 media and growth was followed by measuring OD_580_. (**B**). Growth of H37Rv, *ΔprcBA* and the complemented strains on agar plates. Serial dilutions of the indicated stains were spotted onto 7H11 agar plates and incubated for 3 weeks at 37°C. (**C**) Susceptibility to reactive nitrogen intermediates. Colony forming units (CFU) of H37Rv, *ΔprcBA* and the complemented strains after three days exposure to 0 mM, 3 mM or 5 mM sodium nitrite at pH 5.5. Data are means ± s.d. of triplicate cultures and representative of three independent experiments.

**Figure 5:**
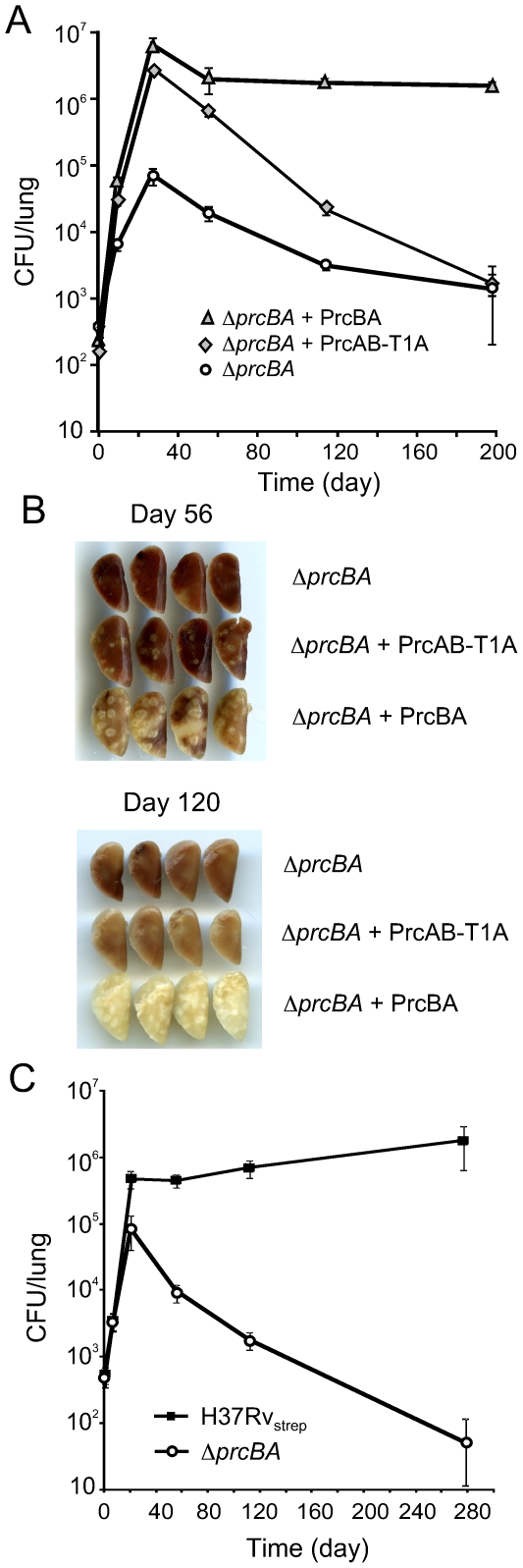
The active site mutant proteasome complements the *in vivo* growth defect but not the persistence defect of the proteasome KO. (**A**) Bacterial titers in lungs of C57BL/6 mice infected by aerosol with *ΔprcBA* and *ΔprcBA* complemented with wild type and the active site T1A mutant ptroteasome. Data are means ± s.d. from four mice per time point per group and represent two independent experiments. (**B**) Gross pathology of lungs infected with *ΔprcBA* and *ΔprcBA* complemented with the wild type and the active site mutant proteasome at day 56 and day 120 post-infection. (**C**) Bacterial titers in lungs of C57BL/6 mice infected by aerosol with a mixed culture of H37Rv with a streptomycin resistance conferring plasmid integrated in the chromosomal attB site (H37Rv_strep_) and the hygromycin resistant *ΔprcBA*.

Surprisingly, expression of PrcAB-T1A in *ΔprcBA* complemented its growth defect both on solid and in liquid media similar to expression of wt PrcBA (Figure 4A, B). The RNI hypersusceptibility of *ΔprcBA* was also complemented to a large degree by the active site mutant proteasome (Figure 4C). We next asked if the catalytic activity of the proteasome is required for *M. tuberculosis* to grow and persist in mice. The mutant proteasome complemented the *in vivo* growth defect of *ΔprcBA* similar to the wt proteasome (Figure 5A), suggesting that a proteolysis-independent activity of the 20S core is required for optimal growth of *M. tuberculosis* in mouse lungs. However, the persistence defect of *ΔprcBA* during the chronic phase of infection was not complemented by the T1A mutant proteasome and bacterial numbers in the lungs declined by 3 log_10_ between day 28 and day 200. Similarly, lung pathology, which was easily detectable on day 56 post-infection, decreased from day 56 to day 200 in mice infected with the T1A mutant proteasome complemented *ΔprcBA* (Figure 5B). Of note, *ΔprcBA* complemented with the mutant proteasome lost viability faster than *ΔprcBA* in mouse lungs (Figure 5A). The higher bacterial burden reached by the strain expressing the mutant proteasome at three weeks post infection likely resulted in a more efficient activation of the immune system resulting in faster killing of the bacilli. This hypothesis is supported by the increased kinetics of killing of *ΔprcBA* in the face of a higher bacterial burden when mice were infected with a mixture of equal numbers of wt *M. tuberculosis* and *ΔprcBA* (Figure 5C).

Altogether, these data suggest that the proteolytic activity of the proteasome is dispensable for growth of *M. tuberculosis in vitro* and in mice and for RNI resistance, yet proteasome mediated proteolysis is required for persistence of *M. tuberculosis* in mouse lungs.

### A proteolytically active proteasome is required for survival in stationary phase and during starvation

**Figure 6:**
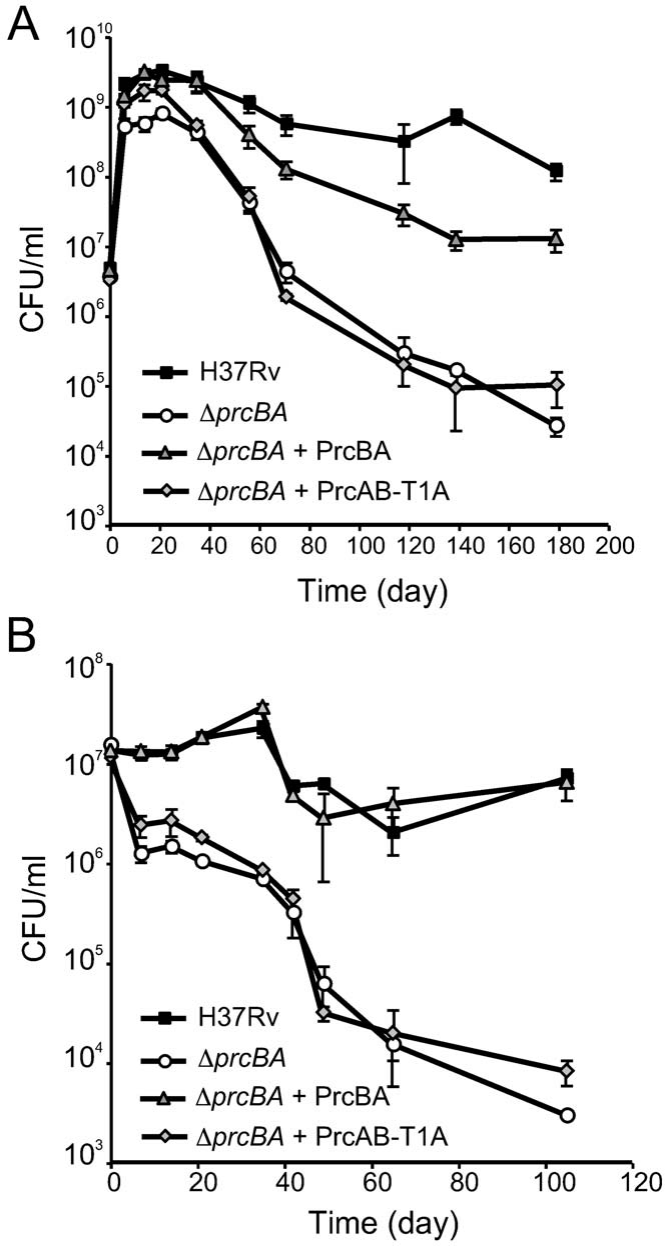
A proteolytically intact core proteasome is required for long-term survival in stationary phase and during starvation. (**A**) Growth and survival of H37Rv, *ΔprcBA* and the complemented strains in complete 7H9 growth medium. (**B**) Survival of H37Rv, *ΔprcBA* and the complemented strains in phosphate buffered saline. CFU were determined at indicated times by plating on 7H11 agar plates. Data are means ± s.d. of triplicates and representative of three independent experiments.

Proteasomal proteolysis may be required during bacteriostasis or periods of slow replication to counter the effects of accumulating protein damage as well as to provide amino acids for energy metabolism during starvation. Proteolysis mediated by ClpP and Lon is important for the ability of *E. coli* to sustain starvation [34], [35]. We monitored survival during stationary phase and during complete starvation of wt *M. tuberculosis*, *ΔprcBA* and *ΔprcBA* complemented with the wt proteasome and the T1A active site mutant proteasome. In normal growth medium, *M. tuberculosis* grew exponentially for about 10 days, after which bacterial numbers remained almost constant over the next 170 days (Figure 6A). *ΔprcBA* was, as expected from earlier growth characterizations, impaired for growth. Upon entering stationary phase, viability of the *ΔprcBA* declined steadily. At 180 days post inoculation there was an approximately 3 log_10_ difference in viable counts between wt and *ΔprcBA* (Figure 6A). Both the growth and persistence defects were largely complemented by expression of the wt proteasome. The T1A mutant proteasome complemented the initial growth defect but failed to complement the persistence defect. Wt *M. tuberculosis* and *ΔprcBA* transformed with the wt proteasome also survived conditions of complete starvation without a significant decline in viability (Figure 6B). In contrast, viability of *ΔprcBA* and *ΔprcBA* transformed with the T1A mutant proteasome declined steadily during starvation (Figure 6B). Collectively, these data indicate that the proteasomal proteolysis is important for the ability of *M. tuberculosis* to survive conditions of starvation and stationary phase *in vitro*.

## Discussion

The eukaryotic proteasome is ubiquitous and essential for many basic cellular processes including differentiation, proliferation, transcription, signal transduction, metabolic regulation, immune surveillance and others [36], [37]. In prokaryotes, proteasome deletion mutants have been generated in *Thermoplasma acipophilum*, *Streptomyces lividans*, *S. coelicolor* and *M. smegmatis*. *T. acidophilum* proteasome mutants were impaired for survival post heat shock but not under normal growth conditions [38]. Deletion of the proteasome in *S. lividans*, *S. coelicolor* and *M. smegmatis* did not reveal phenotypic defects [39], [40], [41]. The current work proves that the 20S proteasome is not essential for growth of *M. tuberculosis*. However, consistent with previous studies [24], [25] lack of the core proteasome resulted in a growth defect on plates and in liquid culture, suggesting that proteasome-mediated proteolysis is important for optimal *in vitro* growth of *M. tuberculosis*.

Surprisingly, however, a proteolytically defective proteasome fully complemented the growth defects and partially restored the RNI hypersusceptibility of *ΔprcBA*. Thus, these phenotpyes of *ΔprcBA* are likely not due to lack of proteasomal proteolysis. We cannot exclude that mutation of the active site threonine to alanine failed to completely abolish proteolysis. However, peptidolytic activity of the proteasome was undetectable in lysates expressing the T1A mutant proteasome and the proteasome substrate PanB tagged with GFP accumulated similarly in *ΔprcBA* and *ΔprcBA* expressing the T1A mutant proteasome. Thus, proteasome-mediated proteolysis was drastically impaired when the active site threonine was mutated to alanine in PrcB. The 20S core requires accessory factors for protein targeting to the proteolytic chamber [42]. In eukaryotes, proteasome accessory factors are not only found as part of the proteasome complex; they also form subcomplexes that regulate transcription and DNA repair and have chaperone function [43], [44], [45]. The stoichiometry of proteasome accessory factors that are free or in complex with the 20S core might be regulated. In the absence of the 20S core an excess of free accessory factors might affect growth. The T1A active-site mutant proteasome is likely to interact with Mpa and potentially other proteasome accessory factors despite its catalytic defect and thereby prevent phenotypes caused by an imbalance of free and complexed accessory factors.

Depletion of the 20S proteasome in *M. tuberculosis* caused hypersusceptibility to RNI and impaired persistence in the mouse [24]. Deletion of Mpa and PafA sensitized *M. tuberculosis* to RNI *in vitro* and resulted in impaired growth in the mouse [8]. However, the proteolytically defective active site mutant proteasome complemented the RNI hypersusceptibility of *ΔprcBA* to a large degree, suggesting that proteasomal proteolysis is not essential for conferring RNI resistance. Moreover, inhibition of iNOS, the major producer of nitric oxide in macrophages, had no impact on survival of *ΔprcBA* in mouse lungs. Thus other factors of the adaptive immune response appear responsible for killing *ΔprcBA* during the chronic phase of the infection. Of note, the attenuated growth of the *M. tuberculosis* Mpa mutant in the acute phase of infection was not rescued in iNOS-deficient mice [8]. Phagocyte oxidase activity may have compensated for inhibited iNOS activity; however, proteasome-depleted *M. tuberculosis* and the Mpa mutant were hyperresistant to oxidative stress *in vitro* [8], [24].

We propose that nutrient limitation might be responsible for the killing of *ΔprcBA* in mice. *M. tuberculosis* lacking the 20S core failed to survive prolonged stationary phase and nutrient starvation *in vitro* and was unable to persist *in vivo*. Proteasome-mediated proteolytic turnover seems essential for *in vitro* and *in vivo* persistence of *M. tuberculosis*, because the active site mutant proteasome expressing strain phenocopied the inability of *ΔprcBA* to persist both *in vitro* and *in vivo*. Nutritionally starved and clinically persistent *M. tuberculosis* share phenotypic similarities, including reduced acid-fastness and drug tolerance [46], [47], [48]. Starvation of *M. tuberculosis* reduced respiration to minimal levels, indicating a low metabolic activity, but the bacilli remained viable and were recoverable when returned to rich medium [49], [50]. Nutrient starvation-induced transcripts can be detected in human tuberculous granulomas [46], [51]. Moreover, the stringent response, required for long-term survival in culture, was also required for persistence of *M. tuberculosis* in mice [52], [53], [54]. In *E. coli* amino acid starvation is followed by increased ribosomal protein degradation via Lon protease to provide amino acids for the synthesis of new enzymes important for adaptation to starvation [35], [55]. Moreover, starvation and growth arrest are linked to the production of misfolded and aberrant proteins isoforms that need to be degraded to prevent toxicity [56]. In mammalian cells proteasomal protein degradation is crucial in supplying amino acids for the synthesis of new proteins during amino acid deprivation [57]. Similarly, *M. tuberculosis* might require the proteasome for amino acid supply and turnover of damaged proteins during long term persistence within its host.

In summary, this work demonstrates the essential role of the 20S proteasome proteolytic activity for *M. tuberculosis* to persist *in vivo* and reveals a mechanism beyond nitric oxide defense by which the proteasome contributes to mycobacterial fitness.

## Materials and Methods

### Ethics statement

All mouse procedures performed in this study were conducted following the National Institutes of Health guidelines for housing and care of laboratory animals and performed in accordance with institutional regulations after protocol review and approval by the Institutional Animal Care and Use Committee of Weill Cornell Medical College.

### Strains, media and culture conditions

Wild-type *M. tuberculosis* (H37Rv) was obtained from Dr. Robert North, Trudeau Institute. Mycobacteria were grown at 37°C in Middlebrook 7H9 medium (Difco) containing 0.2% glycerol, 0.5% bovine serum albumin, 0.2% dextrose, 0.085% NaCl, and 0.05% Tween 80. Hygromycin B (50 mg/ml), kanamycin (15 mg/ml) and streptomycin (20 mg/ml) were included when required for selection.

### Construction of *ΔprcBA*

*PrcBA* genes were deleted from the chromosome via homologous recombination following transduction with temperature-sensitive mycobacteriophage phAE87 [58]. 768 bp upstream of the start codon of *prcB* and 524 bp downstream of the stop codon of *prcA* were amplified by PCR from H37Rv genomic DNA and cloned into pJSC284 to flank the hygromycin resistance gene. pJSC284 is a derivative of pYUB854 containing a lambda cos site, and a unique *Pac*I site. The resulting plasmid was ligated with the temperature-sensitive phage phAE87 and the resulting phage was used to infect *M. tuberculosis*. Hygromycin-resistant transductants were selected on 7H11 agar plates with 50 µg/ml hygromycin for 3 weeks and analyzed by Southern blot.

### Complementation of *ΔprcBA*

*PrcBA* were PCR amplified from H37Rv genomic DNA with a forward primer specific to *prcB* (5′- CGTCCGCGCATGCGTCCAGGAGGGCGGACAG-3′) and a reverse primer specific to *prcA* (5′-GACACGCGTCGGACGTTTAAACTCAGCCCG-3′). The resulting fragment was cloned into an episomal mycobacterial plasmid containing the mycobacterial promoter P_myc1_tetO [59] and a kanamycin resistance gene.

### Construction of T1A proteasome mutant

*PrcA* was PCR amplified using primers 5′- CGGGTGCGCATGCTTTCGGCTCCGAAGGAGGTGAG-3′ and 5′-ACTCAGCCCGACGATTCGCCGTCAGACTGC-3′ resulting in introduction of an *Sph*I site at the 5′ end of *prcA* followed by a synthetic ribosome binding site (RBS). *prcB* was PCR amplified using primers 5′-GGCCACCATTGTCGCGCTGAAATACCCC-3′ and 5′- CGCCTGCTCTGCAGTCAATGATGATGATGATGATGCTTCTCACCGCCATCGGAGCCGAAAGTATCC-3′ from the H37Rv genome resulting in deletion of the 5′ end encoding its pro-peptide, mutation of threonine 1 to alanine and addition of a C terminal hexahistidine tag, followed by a PstI site 3′ of *prcB*-T1AHis_6_ (encoded protein referred to as PrcAB-T1A). For expression of the active proteasome, *prcBA* was amplified from the H37Rv genome using primers 5′- CGTCCGCGCATGCGTCCAGGAGGGCGGACAG-3′ and 5′-GGGGGCCCATCGATCTCTTAATTAAGGTAGAC-3′. The amplified fragments were cloned into an episomal mycobacterial plasmid containing the mycobacterial promoter P_myc1_tetO [59] and a kanamycin resistance gene.

### Construction of PanB-GFP expression vector

The *panB-gfp* fusion was generated by PCR and cloned using the Gateway Cloning Technology (Invitrogen) behind a constitutive promoter into an integrative mycobacterial plasmid containing a streptomycin resistance gene.

### Immunoblots

Cell lysates were prepared by bead-beating the cell pellets in PBS containing protease inhibitor cocktail (Complete Mini, Roche). Clarified cell lysates were filter sterilized by passage through a 0.2 µm filter. 15 µg cell lysates were subjected to SDS-PAGE, followed by transfer to a nitrocellulose membrane. Blots were probed with PrcB-specific and DlaT-specific rabbit sera at 1∶15,000 and 1∶10,000 dilutions in 5% skimmed-milk containing Tris-buffered saline with 0.05% Tween 20 (TBST). To assess PanB-GFP accumulation, blots were probed with anti-GFP antibody (Invitrogen). Secondary antibodies, donkey anti-rabbit (horse radish peroxidase coupled) or LI-COR 800 goat anti rabbit were used at 1∶30,000 dilution in 2% skimmed-milk containing TBST and at 1∶15,000 dilution in Odyssey blocking buffer, respectively. Blots were developed using Immobilon Western Chemiluminescent HRP substrate (Millipore) or using the Odyssey Infrared Imaging System (LI-COR Biosciences).

### Proteasome activity assay

Bacteria were grown to OD_580nm_ 1.0, washed as described above and cell pellets were lysed in 450 µl PBS containing protease inhibitor cocktail (Complete Mini, Roche) using a bead beater. Clarified cell lysates were filter sterilized by passage through a 0.2 µm filter and then adjusted to a final glycerol concentration of 10%. Proteasome activity was assessed as previously described [12]. Briefly, 50 µg of lysate were incubated with 100 µM Succinyl-Leu-Leu-Val-Tyr-aminomethyl coumarin (Suc-LLVY-AMC) in 20 mM HEPES, 0.5 mM EDTA buffer and fluorescence was monitored at excitation of 370 nm and emission of 430 nm at 37°C over 60 min in a 96 well-plate fluorimeter (Molecular Devices).

### Determination of in vivo GFP and PanB-GFP accumulation

Cultures were grown to OD_580nm_ 0.4–0.9 (mid log) and 1.0–1.5 ml of cultures were harvested, resuspended in 100 ml PBS and aliquoted into a black 96 well plate. Fluorescence was measured using excitation at 485 nm and emission at 515 nm. Relative fluorescence units were normalized to OD_580nm_.

### *In vitro* stress susceptibility assays

Cultures were grown to log phase (OD_580nm_ 0.6), washed in growth medium and single cell suspensions prepared in assay medium by centrifugation at 800 rpm for 12 minutes. Single cell suspensions were subsequently diluted to OD_580nm_ 0.01. To test susceptibility to RNI, diluted cultures were incubated at pH 5.5 with or without 3mM or 5mM NaNO_2_ for 3 days at 37°C. To determine viability, serial dilutions of cultures were plated on 7H11 plates.

### Stationary phase survival and starvation assays

For long-term survival experiments *M. tuberculosis* strains were grown in 7H9 medium to mid log phase. Single cell suspensions were prepared in 7H9 as described above and diluted into fresh medium to OD_580n_ 0.01, in 10 ml triplicate cultures. For starvation conditions, single cell suspensions were prepared in PBS with 0.02% Tween 80 and diluted into PBS-Tween to OD_580n_ 0.01 in triplicate 10 ml cultures. Cultures were incubated at 37°C under constant shaking (50 rpm). To determine growth and viability, serial dilutions of cultures were plated on 7H11 plates.

### Animal infections

Eight week old, female C57BL/6 mice (Jackson Laboratory) were infected with *M. tuberculosis* strains by aerosol as described [24]. Bacterial numbers in organs were enumerated by plating organ homogenates for colony forming units (CFU) at indicated times. N6-(1-Iminoethyl)lysine (NIL; L- and D-enantiomers) (custom synthesized by DeCODE Chemicals [60], and a kind gift from Dr. C. Nathan) were given in acidified (pH 2.7) drinking water (4 mM) beginning on day 21 post infection and freshly prepared every 48 hr until the end of the experiment [26].

## Supporting Information

Figure S1 Genetic deletion of *prcBA*. (A) Map of the *prcBA* genomic region in wt *M. tuberculosis* (top) and Δ*prcBA* (bottom). Probe location and *Bam*HI (B) restriction sites are indicated. (B) Southern blot of *Bam*HI (B) digested genomic DNA from wt *M. tuberculosis* (H37Rv) and Δ*prcBA* probed with the DNA fragment indicated in (A). (C) PrcB levels analyzed by immunoblot in H37Rv, Δ*prcBA* and the complemented mutant (Δ*prcBA* + PrcBA). Dihydrolipoamide acyltransferase (DlaT) was used as loading control.
(0.80 MB TIF)

Figure S2 PrcBA complex formation. (A) Immunoprecipitation of PrcBHis6 and PrcB-T1AHis6 co-purified PrcA. Cell lysates from 100 ml cultures (20 mg total protein) of the indicated *M. tuberculosis* strains were immunoprecipitated with anti-histidine beads (Invitrogen). Protein eluted from the beads (E) with 1 M imidazol and protein recovered from boiled beads (B) were separated on a 15% SDS Page. (B) Identification of PrcB and PrcA by peptide mass finger printing and N-terminal sequencing. N-terminal sequencing of proteins from band 2 and 4 identified the expected N-terminal threonine for PrcB in band 2 and an N-terminal alanine residue for PrcB-T1A in band 4.
(0.31 MB PDF)
