# Supplementary figures and images for: The Mycobacterium tuberculosis Proteasome Active Site Threonine Is Essential for Persistence Yet Dispensable for Replication and Resistance to Nitric Oxide
Source: PLoS Pathog. 2010 Aug 12;6(8):e1001040. doi: 10.1371/journal.ppat.1001040 (PMC2920845; doi:10.1371/journal.ppat.1001040)

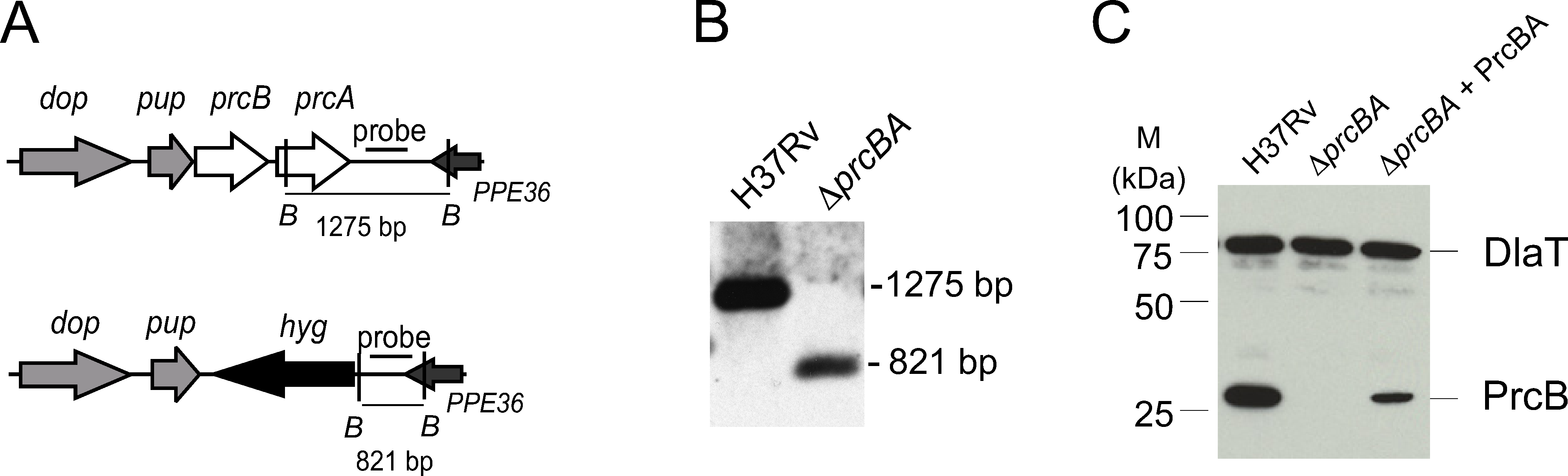

Supplement: Figure S1 — Genetic deletion of prcBA. (A) Map of the prcBA genomic region in wt M. tuberculosis (top) and ΔprcBA (bottom). Probe location and BamHI (B) restriction sites are indicated. (B) Southern blot of BamHI (B) digested genomic DNA from wt M. tuberculosis (H37Rv) and ΔprcBA probed with the DNA fragment indicated in (A). (C) PrcB levels analyzed by immunoblot in H37Rv, ΔprcBA and the complemented mutant (ΔprcBA + PrcBA). Dihydrolipoamide acyltransferase (DlaT) was used as loading control. (0.80 MB TIF) [file ppat.1001040.s001.tif]
