# Supplementary material for: The Mycobacterium tuberculosis Proteasome Active Site Threonine Is Essential for Persistence Yet Dispensable for Replication and Resistance to Nitric Oxide
Source: PLoS Pathog. 2010 Aug 12;6(8):e1001040. doi: 10.1371/journal.ppat.1001040 (PMC2920845; doi:10.1371/journal.ppat.1001040)
Supplement: Figure S2 — PrcBA complex formation. (A) Immunoprecipitation of PrcBHis6 and PrcB-T1AHis6 co-purified PrcA. Cell lysates from 100 ml cultures (20 mg total protein) of the indicated M. tuberculosis strains were immunoprecipitated with anti-histidine beads (Invitrogen). Protein eluted from the beads (E) with 1 M imidazol and protein recovered from boiled beads (B) were separated on a 15% SDS Page. (B) Identification of PrcB and PrcA by peptide mass finger printing and N-terminal sequencing. N-terminal sequencing of proteins from band 2 and 4 identified the expected N-terminal threonine for PrcB in band 2 and an N-terminal alanine residue for PrcB-T1A in band 4. (0.31 MB PDF) [file ppat.1001040.s002.pdf]

A

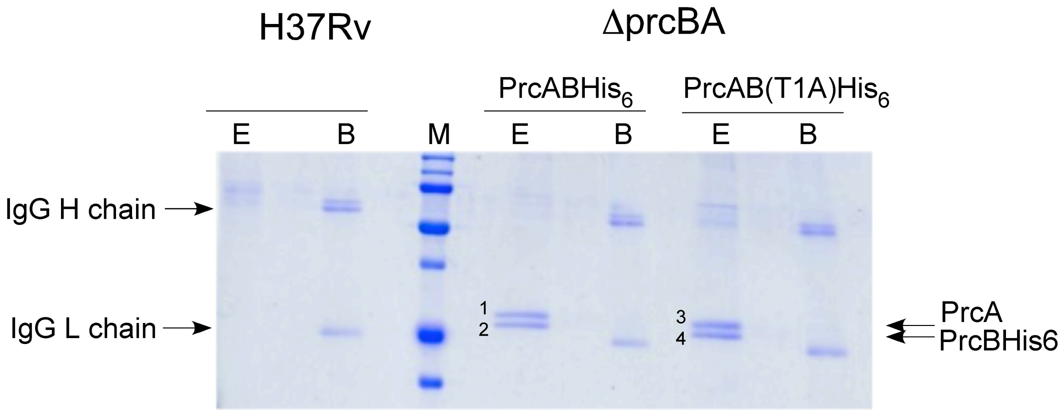

B

Protein ID	Gel band #	Sequence coverage	Sequence (matched peptides in blue)
PrcA	1	42%	1 SFPYFISPEQAMRERSELARKGIARAKSVVALAYAGGVLFVAENPSRSL<br>51 QKISELYDRVGFAAAGKFNEFDNLRRGGIQFADTRGYAYDRRDVTGRQLA<br>101 NVYAQTLGTIFTEQAKPYEVELCVAEVAHYGETKRPELYRITYDGSIADE<br>151 PHFVVMGGTTEPIANALKESYAENASLTDALRIAVAALRAGSADTSGGDQ<br>201 PTLGVASLEVAVLDANRPRRAFRRITGSALQALLVDQESPQSDGESSG
PrcB	2	54%	1 TTIVALKYPGGVVMAGDRRSTQGNMISGRDVRKVYITDDYTATGIAGTAA<br>51 VAVEFARLYAVELEHYEKLEGVPLTFAGKINRLAIMVRGNLAAAMQGLLA<br>101 LPLLAGYDIHASDPQSAGRIVSFDAAGGWNIEEEGYQAVGSGSLFAKSSM<br>151 KKLYSQVTDGDSGLRVAVEALYDAADDDSATGGPDLVRGIFPTAVIIDAD<br>201 GAVDVPESRIAELARAIIESRSGADTFGSDGGEK
PrcA	3	49%	1 SFPYFISPEQAMRERSELARKGIARAKSVVALAYAGGVLFVAENPSRSL<br>51 QKISELYDRVGFAAAGKFNEFDNLRRGGIQFADTRGYAYDRRDVTGRQLA<br>101 NVYAQTLGTIFTEQAKPYEVELCVAEVAHYGETKRPELYRITYDGSIADE<br>151 PHFVVMGGTTEPIANALKESYAENASLTDALRIAVAALRAGSADTSGGDQ<br>201 PTLGVASLEVAVLDANRPRRAFRRITGSALQALLVDQESPQSDGESSG
PrcB-T1A	4	80%	1 ATIVALKYPGGVVMAGDRRSTQGNMISGRDVRKVYITDDYTATGIAGTAA<br>51 VAVEFARLYAVELEHYEKLEGVPLTFAGKINRLAIMVRGNLAAAMQGLLA<br>101 LPLLAGYDIHASDPQSAGRIVSFDAAGGWNIEEEGYQAVGSGSLFAKSSM<br>151 KKLYSQVTDGDSGLRVAVEALYDAADDDSATGGPDLVRGIFPTAVIIDAD<br>201 GAVDVPESRIAELARAIIESRSGADTFGSDGGEK
